# Chronic Idiopathic Thrombocytopenia Purpura and *Helicobacter pylori* Eradication: A case study

**DOI:** 10.4021/gr2009.02.1271

**Published:** 2009-01-20

**Authors:** Santosh K. Tiwari, Manoj G, Aleem A. Khan, Aejaz Habeeb, Chittoor M. Habibullah

**Affiliations:** a Center for Liver Research and Diagnostics, Deccan College of Medical Sciences, Kanchanbagh, Hyderabad 500 058, Andhra Pradesh, India

**Keywords:** Idiopathic thrombocytopenic purpura, *Helicobacter pylori*, Eradication, Platelet

## Abstract

Idiopathic thrombocytopenic purpura (ITP) is an immune-mediated thrombocytopenia. ITP is the result of accelerated platelet destruction by the reticuloendothelial system, primarily the spleen. The prevalence of *Helicobacter pylori* infection and the effect of its eradication were monitored in an ITP patient over a period of 12 months. Eradication of *Helicobacter pylori* led to the increased platelet count and provides a new insight for a non-immunosuppressive treatment in selective ITP patients.

## Introduction

The idiopathic thrombocytopenic purpura (ITP), first described by P.G. Werlhof, is characterized by premature destruction of autoantibody-coated platelets [1], causing thrombocytopenia and subsequent mucocutaneous bleeding. The gastric pathogen *Helicobacter pylori* (*H. pylori*) has been recognized in the etio-pathogenesis of gastritis, peptic ulcer and also implicated with gastric adenocarcinoma and mucosa-associated lymphoid tissue (MALT) lymphoma. Several studies have proposed that *H. pylori* infection may be associated with numerous conditions, including pernicious anemia, autoimmune neutropenia, membranous nephropathy, autoimmune thyroid disease, and immune thrombocytopenic purpura [2, 3, 4].

We report a case of 40-year-old woman with ITP who experienced a complete and lasting normalization of the platelet count after receiving treatment for the eradication of *H. pylori* infection.

## Case Report

A 40-year-old lady with untreated chronic ITP was admitted in Owaisi hospital and Research Center, Hyderabad, India. She suffered from bleeding gums with the appearance of generalized purple spots all over the body, bleeding into the right eye and melena since last 10 days. It was not associated with fever but had one episode per day of severe abdominal pain with vomiting. Retrospection of her past history revealed similar kind of attacks in the past 7 years and this was her third episode. An upper gastrointestinal endoscopy showed fundal and corpus hemorrhagic gastritis and biopsy test for the presence of *H. pylori* infection confirmed active infection by mucosal polymerase chain reaction. Other parameters of the patient upon investigation on the first day showed blood pressure of 90/50mm Hg with a platelet count of 40 x 10^3^/mm^3^ and hemoglobin was found to be 3g%. The patient was conservatively managed by giving 2 units of packed cells followed by 2 units of platelet with plasma for 4 days, owing to the positivity to *H. pylori* infection, the patient was kept on oral Pantaprazole (40mg, twice daily), amoxicillin + clavulanate (625mg, twice daily) along with oral Clarithromycin (500mg, twice daily) for 7 days; the patient was also given Cifran eye drops (quarter in die).

The patient was prescribed oral wysolone (10mg, once daily) initially. Later withdrawal of wysolone resulted in the relapse (steroid dependant thrombocytopenia) of another episode of melena. The patient was discharged on the eleventh day with a platelet count of 90 x 10^3^/mm^3^, hemoglobin 6.2 g%, blood pressure was 110/80mm Hg. The patient continued to take Pantaprazole orally (40mg twice daily) for 6 weeks and was advised to use wysolone (S.O.S). The patient continued to take steroids for a period of 1 month in tapered doses (once the alternate day) and later discontinued (after one month), however, she continued to take pantaprazole to complete the 6 weeks course. The platelet count of the patient was monitored every fortnight and was found to be normal after completion of one-week anti-*H. pylori* therapy and six weeks of PPI intake. Endoscopy was performed again at the end of the treatment, which showed reduction in the fundal gastritis and absence of *H. pylori* infection by PCR and histology, though it showed a polyclonal B lymphocytic infiltration of the gastric mucosa. A control endoscopy performed along with platelet count after 12 weeks of the eradication therapy showed normal fundus with no evidence of ulceration in any part of the stomach, and platelet count was in the normal range. The patient no longer continued steroid intake after one month. The patient’s platelet count was again assessed after 12 months of the therapy and was found to be 160 x 10^3^/mm^3^.

## Discussion

The first case of association of *H. pylori* with ITP was reported in 1998 [5]. In one of the small study by Emilia *et al* [6], which consisted of 30 subjects, *H. pylori* positivity was found in 13 subjects. Bacterium eradication with antibiotics, in 12 of 13 infected patients (92.3%), led to a complete response in 4 (33.33%) and to a partial response (platelets 90 - 120 x 10^3^/mm^3^) in 2 (16.66%) [6]. Many hypotheses have suggested that correlate *H. pylori* infection with thrombocytopenia. Some profess that *H. pylori* initiates autoreactivity through molecular mimicry, another class of experts recognize that as *H. pylori* express Lewis antigens in a strain-specific manner; Lewis antigens adsorb to platelets and might serve as targets for anti-Lewis antibodies in patients with an appropriate genetic background [7]. In addition, both *H. pylori* infection and ITP are associated with a T helper 1 (Th1)–type immune response characterized by increased levels of interferon-γ and interleukin-2; hence, *H. pylori* associated increase of the cytokine profiles may contribute to the development of immune thrombocytopenia. In addition to this, few studies have also demonstrated a temporal association between the disappearance of anti-CagA antibodies in the serum and improvement of ITP [8]. Co-incidently, the *H. pylori* strain in our case was also found to be *cag*A positive along with *cagE*, *vac*As1 positivity. However, consistent with the findings of Michel *et al* [9], others have reported neither an increased incidence of *H. pylori* in patients with ITP nor improvement in platelet counts after eradication [9]. The reason for these discordant results is uncertain but may reflect the results of studying diverse patient populations, failing to control for administration of concomitant therapies, or variable effects of genetically diverse *H. pylori* strains in remote geographic regions. Furthermore, our study for the first time demonstrated *H. pylori* infection using PCR in contrast to other studies which had used serological assays or histopathological tests to confirm this infection. Initial treatment of the patient with steroids was mandatory so as to rescue the patient from the critical condition and should not be a limitation of the report, as later it is seen that patient platelets did not decrease at tapered doses and even after stopping it completely.

In conclusion, eradication of *H. pylori* infection led to a good platelet response in our chronic ITP patient. It is therefore hypothesized that eradication of *H. pylori* infection in such patients may alleviate the use of immunosuppressive agents in combating hematological disorder like ITP. At the same time, it is also important to initiate studies in broad number of subjects from different geographical areas with probably longer follow-up, so as to evaluate the molecular pathway that links *H. pylori* with ITP. Studies of similar type may also be helpful in assessing the long lasting effect of anti-*H. pylori* therapy in patients with chronic ITP.
